# Development of competencies in secondary education through the motivational style of autonomy support

**DOI:** 10.12688/f1000research.144919.1

**Published:** 2024-03-07

**Authors:** Miguel Llorca-Cano, Juan Antonio Moreno-Murcia, Julio Barrachina-Peris, Elisa Huéscar

**Affiliations:** 1 Deporte y salud, Universidad Miguel Hernandez de Elche, Elche, Valencian Community, 03202, Spain; 2 Educación, Universitat d'Alacant, Alicante, Valencian Community, 03690, Spain

**Keywords:** motivation, basic psychological needs, active methodologies, learning, commitment.

## Abstract

**Background:**

The aim of the study was to test the effect of a meta-disciplinary intervention based on the motivational style of autonomy support on the development of competencies in secondary school students. It was carried out by means of a quasi-experimental design and lasted for three months.

**Methods:**

A total of 62 students between the ages of 12 and 16 (M = 13.61; SD = 1.16) participated, with 33 in the experimental group and 29 in the control group, along with 12 teachers (7 in the intervention group and 5 in the control group). The study measured teaching motivational style, satisfaction of basic psychological needs, motivation, and key competencies.

**Results:**

The results demonstrate improvements in the autonomy-supportive motivational style, satisfaction of the basic psychological need for autonomy, autonomous motivation, and competencies in the experimental group, while the control group exhibited an increase in the chaos style.

**Conclusions:**

These findings reveal the positive impact of the supportive motivational style on the development of key competencies establishing it as an active, valid, and reliable methodology to motivate secondary school students.

## Introduction

The data and figures published by the Ministry of Education and Vocational Training on educational outcomes ( MEFP, 2021) position Spain with the highest repetition rate in Compulsory Secondary Education (ESO) in the European Union and with an early school dropout rate (not concluding ESO) only surpassed by Malta. The PISA Report (2018) revealed that students with strong attachment to their school received greater emotional support from their families and were less likely to be absent from school.

The analysis of school climate found that most students considers very positive to help their peers, and more than a third said that Spanish teachers waited a long time for classmates to calm down. Students scored higher in reading when they perceived their teachers to be enthusiastic, to show interest in the subject and to enjoying teaching. These data suggest that creating a learning climate in which students feel motivated to complete academic tasks is now a priority for teachers.

Recent studies show the relationship between the autonomy-supportive motivational style and a learning climate that fosters student involvement through its impact on mediators, regardless of the content addressed ( Cheon, Reeve and Vansteenkiste, 2020; Gómez Rijo *et al.*, 2023; Niemiec and Ryan, 2009; Reeve and Cheon, 2021; Reeve and Shin, 2020; Ryan and Deci, 2020). Thus, the use of a positive communicative style in class, (e.g. autonomy support), which extols the value of the student’s personal progress and is accompanied by explanations, has been shown to positively predict academic performance and task engagement, as these messages satisfy basic psychological needs ( Santana-Monagas *et al.*, 2021, 2022a, 2022b).

Current research on classroom climate is adopting a new perspective supported by a multidimensional or circumplex approach ( Aelterman *et al.* 2019; Escriva-Boulley *et al.*, 2021; Cohen *et al.*, 2022; Moé *et al.*, 2022; Vermote *et al.*, 2020, 2022), with the aim of interpreting more accurately the relationships between oscillations in student-perceived motivational climate and changes in teacher motivational style.

**Figure 1.**
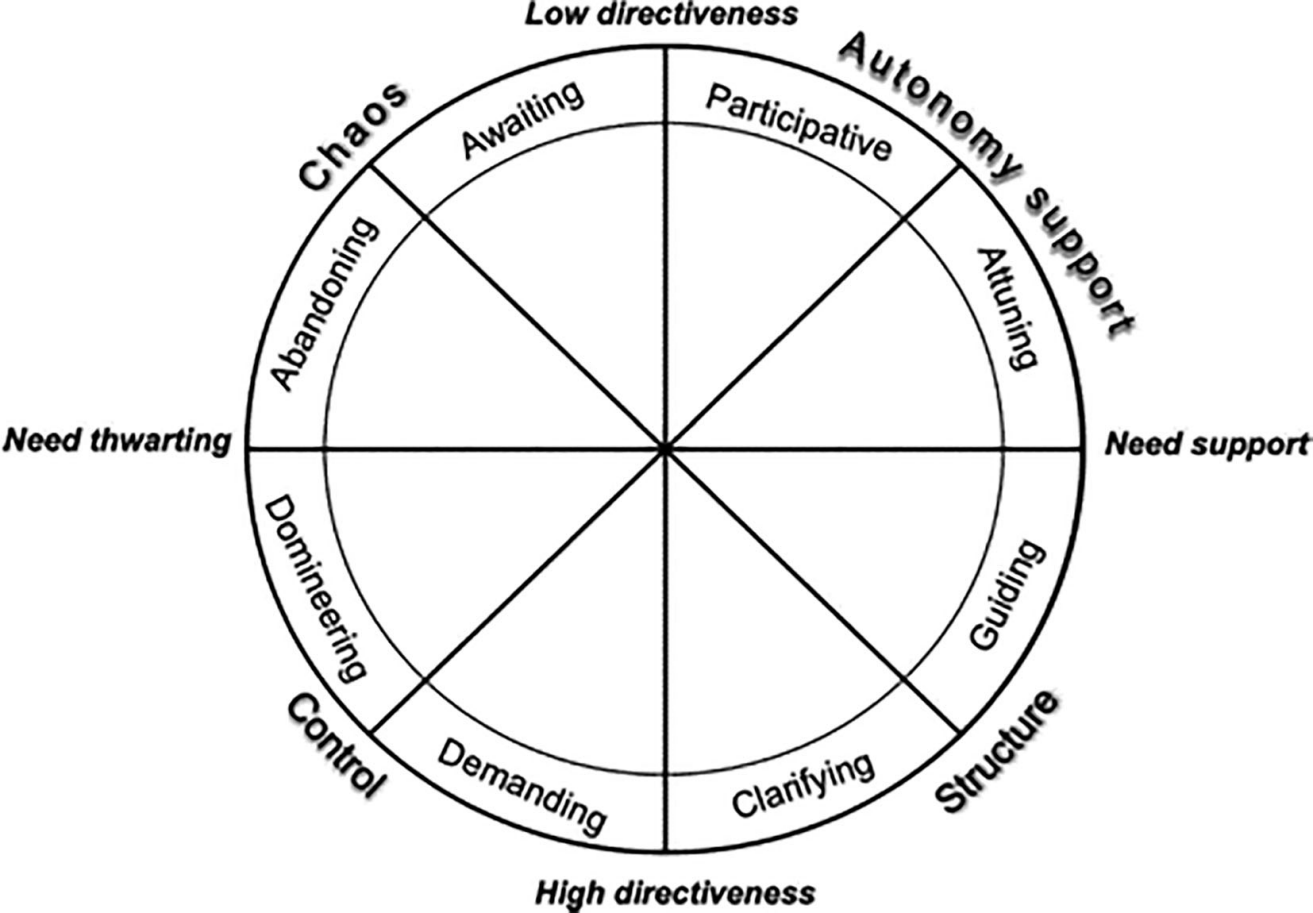
Circumflex model ( Aelterman *et al.*, 2019).

In summary, the circumplex model ( Aelterman *et al.* 2019) adopts a multidimensional structure based on the degree of support or frustration of basic psychological needs and the greater or lesser directivity of the teaching intervention, integrating four major styles. Two of them are on the axis of basic psychological needs (Control and Chaos vs Autonomy Support and Structure) and the other two on the directivity axis (Control and Structure vs Chaos and Autonomy Support). These styles are concretized around eight subdimensions and associated in pairs to the styles: autonomy support (participative and attuned) and structure (guiding and clarifying) on the basic psychological-needs axis, and chaos (waiting and abandoning) and control (demanding and demanding) on the directivity axis ( Figure 1).

The model has been implemented through the Situations-in-School (SIS) questionnaire ( Aelterman *et al.*, 2019), enabling the simultaneous analysis of the effect of the four styles and their adjacencies in different contexts ( Burgueño *et al.*, 2023; Cohen *et al.* 2022; Delrue *et al.* 2019; Escriva-Boulley *et al.*, 2021; Franco *et al.*, 2023; Van Doren *et al.*, 2023). It also provides evidence that when teacher intervention during instruction is adaptive, learning demands are more effectively met and quality motivation develops ( Van Doren *et al.*, 2023).

In this sense, given the affinities between the demands of competency learning and the strategies presented by an adaptive motivational style (Autonomy Support and Structure) ( Hamodi, Moreno-Murcia, and Barba, 2018; Moreno-Murcia, Llorca-Cano, and Huéscar, 2020; Moreno-Murcia, Ruiz, and Vera, 2015), it is expected that this approach can help boost the development of students’ competencies, as postulated in the educational system ( LOMLOE, 2020). The literature has shown that adequate student functioning in class is related to the optimization of their autonomy, through the support of student autonomy ( Pérez-González *et al.*, 2019; Van Doren *et al.* 2023) and that such functioning requires satisfying the mediators (autonomy, competence, and relationship with others), bringing numerous benefits at all levels (cognitive, behavioural and emotional) ( Ryan and Deci, 2020; Tian and Shen, 2023).

Competency-based learning ( Bolívar, 2010; Valle and Manso, 2013) emphasizes student involvement in terms of performance or performance requirements, giving relevance to autonomy, reflection, and responsibility. It also emphasizes the importance of the teacher as a mediator or facilitator in the process of acquisition and development of the competencies established in the exit profile ( Royal Decree, 217/2022). Thus, since the development of competencies requires the activation of adaptive behavioural patterns, in terms of performances with progressive autonomy, the teacher’s intervention should be adjusted to the demands posed by the process. This involves supporting the student’s autonomy, clarifying, and guiding their learning, empathizing, and providing emotional support ( Zang, 2022) to generate a positive classroom climate that stimulates their involvement and, therefore, motivates them to continue learning.

In this direction, Gómez Rijo *et al.* (2023) conclude that strategies such as the promotion of student participation in the evaluation and the establishment of standards, peer learning, or the design and co-direction of tasks are methodological alternatives that encourage the support of student autonomy in the classroom, as previously demonstrated by other studies ( Cheon, Reeve and Vansteenkiste, 2020; Cents-Boonstra *et al.*, 2022; Reeve and Shin, 2020). These findings demonstrate that employing learner-centered methodologies predicts autonomous motivation and improves learner engagement in class ( Leo *et al.* 2020; Tian and Shen, 2023).

However, despite the existing synergies between the autonomy-supportive motivational style and the development of competencies, there are hardly any studies that provide evidence of the impact of an intervention based on the autonomy-supportive motivational style on the development of competencies ( Barrachina, 2017; Barrachina-Peris *et al.*, in press). Considering the importance acquired by competencies in the educational profile of 21 ^st^-century students ( European Commission, 2018; Royal Decree, 2017/2022) and the relevance given to teachers in this process, the present study is proposed. Its main purpose was to test the effect of the motivational style of autonomy support on the development of competencies, Basic Psychological Needs satisfaction and autonomous motivation.

### Self-determined motivation

Deci Ryan’s (2000, 2002) Self-Determination Theory (SDT) aims to explain human behaviour through various motivational styles, contextual influences, and interpersonal perceptions. According to SDT, there are three basic psychological needs directly linked to motivation and personal well-being: autonomy, which refers to the level of independence and control that a person experiences over his or her decisions (feeling an active part); competence, related to the ability to feel capable of successfully developing a task (perception of self-efficacy); and relatedness to others, associated with the establishment of socio-affective bonds with peers (developing a group identity) ( Ryan and Deci, 2017).

Motivation, according to Ryan and Deci (2017), can manifest itself in different gradients that fluctuate based on the degree of self-determination (intrinsic motivation, extrinsic motivation, and demotivation).

In this sense, if a teacher can provide a context that encourages active involvement in decision-making among students, focuses on the process rather than the outcome and acts as a facilitator, students will develop a more self-determined motivational orientation towards the content presented in class ( Moreno-Murcia *et al.*, 2012). For this reason, interventions that guide teachers towards improving their interpersonal style are considered fundamental.

### Autonomy support

The motivational teaching style can influence the motivation of students during their classes and can be situated along a spectrum ranging from a controlling style to the support of student autonomy ( Tessier *et al.*, 2010). Regarding the latter, the aim is to satisfy the three basic psychological needs and consequently, achieve self-determined motivation. On the contrary, extreme control, which relies on pressuring students without their active participation in the process, may lead them to act from a more extrinsic perspective ( Cheon and Reeve, 2015; Escriva-Boulley *et al.*, 2018; Fin *et al.*, 2019; Haerens *et al.*, 2015; Reeve, 2010; Yew and Wang, 2016).

There is evidence that using an autonomy-supportive interpersonal teaching style can be an effective trigger for the development of intrinsic motivation, which is the most valuable for learning as it elicits student engagement based on interest, enjoyment, and willingness to learn ( Chang *et al.*, 2016; Moreno-Murcia *et al.*, 2012; Ntoumanis and Standage 2009). A teacher’s ability to foster self-determined motivation in their students is crucial for achieving goals set ( Taylor *et al.*, 2010). As demonstrated by Reeve (2016), the use of an autonomy-supportive interpersonal style by the teacher promotes a classroom climate in which students become more proactive, d increase their commitment to the task, and take more responsibility in the learning process as they have a more prominent role ( Hamodi *et al.*, 2018; Moreno-Murcia *et al.*, 2020).

### Competency-based education and motivation

Today, a versatile educational paradigm is emerging, structured around key and life-long competencies ( OECD, 2005). A competency is demonstrated when an action results from reflection and the appropriate application of practical skills, knowledge, motivation, values and attitudes required by the task itself ( OECD, 2002, 2005). Bolívar (2010) states that the competency-based approach represents a paradigm shift compared to previous models that were based on the fragmentation, accumulation and reproduction of knowledge. Itgives importance to the mobilization of knowledge in a specific context and the autonomy of students to manage their own learning. In this approach, according to Royal Decree 217/2022 of March 19, the role of the teacher is fundamental in the transfer process, as they act as a facilitators of learning by using active, contextualized methodologies, and maintaining levels of motivation in students. Such approach promotes meaningful and functional learning prioritizes the applicability of knowledge, its transfer to different contexts and emphasizes a globalizing and transversal approach (order ECD/65/2015; Bolivar, 2010; OECD, 2005). Even though it has become an entrenched model ( LOE, 2006; LOMCE, 2013; LOMLOE, 2020), difficulties are still encountered in its practical implementation ( Pérez-Pueyo *et al.*, 2013; Barrachina and Blasco, 2012; Zapatero-Ayuso *et al.*, 2017). In this context of change, there is a need for successful initiatives aimed at the development of key competencies in students. Principles such as self-regulation, autonomy and interaction underlie the competency-based approach and have been widely addressed in the Self-Determination Theory (SDT) ( Deci and Ryan, 2000). Supported by this theory, different studies have described the positive relationship between the interpersonal style of autonomy support and student involvement, interest, and enjoyment in their classes, as well as the development of a favorable classroom climate and more meaningful and functional learning ( Moreno-Murcia and Sánchez-Latorre, 2016; Fin *et al.*, 2019; Jang *et al.*, 2016). Research has shown that the nature of teacher optimization on student motivational factors is a crucial factor in improving educational quality and student success ( De Jong *et al.*, 2022; Monarca and Rappoport, 2013; Schuster *et al.*, 2021; Hargreaves, 2019).

So far, there have been limited studies that have evaluated the impact of collaborative interventions on key competencies. Additionally, it is also challenging to find studies that analyse the relationship of these competencies with other variables that can predict active and self-determined learning such as the motivational teaching style ( Barrachina-Peris, 2017; Moreno-Murcia *et al.*, 2020).

## Method

### Participants

The sample consisted of 62 Spanish students from compulsory secondary education with ages between 12 and 16 years old (M = 13.61; SD = 1.16). The female to male ratio was 50/50:, female (n = 31) and male (n = 31). They were divided into an intervention group (n = 33), of which 18 were boys and 15 girls, and a control group (n = 29), composed of 13 boys and 16 girls. On the other hand, 12 teachers between 28 and 57 years of age (M = 40.66; SD = 10.50) participated, 58% of whom were male (n = 7) and 42% female (n = 5). The participating teachers were divided into two groups, experimental (n = 7) and control (n = 5).

### Measures

**Autonomy support.** The Situations-in-School (SIS) questionnaire ( Aelterman *et al.*, 2019), validated in the Spanish context by Moreno-Murcia *et al.* (2023) and composed of a total of 60 items, was used. This questionnaire determines the interpersonal style used by the teacher, looking at how the teacher acts in 15 possible situations or scenarios that occur during the sessions and, in turn, 4 ways of acting are presented for each of these situations (one for each teaching style: autonomy support, structure, control and chaos). Therefore, there would be 15 situations with 4 ways of resolution, totalling 60 answers when completing the questionnaire (e.g. for autonomy “When presenting the rules in class, the teacher invites us students to give our opinions about the rules, so that they help us feel comfortable in class”; for control “the teacher tells us students that we must follow them all as he says, even warning us that there will be sanctions if we do not comply”; for structure “the teacher announces his expectations to start cooperating with us”; for chaos “the teacher does not care at all about the rules or our opinions”). It was measured through a Likert-type scale ranging from 1 ( *does not describe me at all*) to 7 ( *describes me extremely well*). Cronbach’s Alpha for the pre-test and post-test of the different dimensions that make up the questionnaire were as follows: autonomy (.89 and .90), structure (.85 and .88), control (.83 and .88) and chaos (.87 and .90).

**Basic psychological needs in the academic environment.** The Spanish translation of the Échelle de Satisfacción des Besoins Psychologiques in the educational context ( León *et al.*, 2011) by Gillet *et al.* (2008) was used. This scale consists of 15 items to measure three dimensions: perception of autonomy (e.g. “I have a say in the development of the programs of my subjects.”), perception of competence (e.g. “I often feel that I can do well.”) and perception of relatedness (e.g. “I feel at ease with others.”). Responses are rated on a Likert-type scale from 1 ( *strongly disagree*) to 5 ( *strongly agree*) points. The Crombach’s Alpha of the different dimensions in the pre-test and post-test of the intervention were in: Autonomy (.75 and .76), competence (.77 and .77), and relatedness (.80 and .79).

**Academic motivation.** The Spanish version of the Échelle de Motivation en Éducation, Escala de Motivación Académica (EMA) by Núñez, Martín-Albo and Navarro (2005) was used. The EMA is composed of 28 items distributed in seven subscales of four items each: demotivation (e.g. “I don’t know why I go to high school and, honestly, I don’t care.”), external regulation (e.g. “Because I want to *live well* once I finish my studies.”), introjected regulation (e.g. “Because it will help me make a better decision regarding my career direction.”), identified regulation (e.g. “Because when I do well in class I feel important.”), intrinsic knowledge motivation (e. g. “Because my studies allow me to continue learning many things that interest me.”), intrinsic achievement motivation (e.g. “Because of the satisfaction I feel when I overcome difficult academic activities.”) and intrinsic motivation to stimulating experiences (e.g. “Because for me, high school is fun.”). They were preceded by the pre-question “Why do you study this subject?”, and were measured using a Likert-type scale ranging from 1 ( *Does not correspond at all*) to 7 ( *corresponds completely*). The consistency of each dimension was for: autonomous motivation (.94 in the pre-test and .93 in the post-test) and for controlling motivation (.78 in the pre-test and .75 in the post-test). Intrinsic motivation to knowledge (.82 at pre-test and .79 at post-test), intrinsic motivation to achievement (.82 at pre-test and .87 at post-test), intrinsic motivation to stimulating experiences (.82 at pre-test and .76 at post-test), identified regulation (.80 at pre-test and .75 at post-test), introjected regulation (.78 at pre-test and .75 at post-test), external regulation (.85 at pre-test and .85 at post-test), and demotivation (.90 at pre-test and .90 at post-test).

**Student competences.** The “Key Competences Perception Scale” (ECC) developed by Moreno-Murcia *et al.* (2015) was used, which is composed of 9 items that measure the students’ perception of the acquisition of the different key competences. These items (e.g. “Expressing my ideas and respecting those of others”) are preceded by the previous sentence “What my teacher is teaching me allows me to be able to…”. Responses were rated by means of a Likert scale ranging from 1 ( *Strongly disagree*) to 7 ( *Strongly agree*). The internal consistency of this dimension was .88 in the pre-test and .84 post-test.

### Design and procedure

The project was approved by the Project Evaluation Body of the Miguel Hernández University (2017.06.259.E.OEP). This study was developed according to the principles expressed in the Declaration of Helsinki; First, the school administration was contacted to explain the objective of the study, and participation was approved through the school council. The parents/guardians of the students were asked for written authorization to participate and the treatment of the data was guaranteed in accordance with the institutional ethical guidelines regarding consent, confidentiality and anonymity. Questionnaires were completed at the beginning and end of the intervention.

A quasi-experimental design was used for sample selection, given that the participants could not be randomly selected because they were previously divided into groups. The sample was distributed into 13 groups, of which 7 had a teacher who followed an intervention model with a motivational style of autonomy support and the remaining 6 did not follow differentiated methodological guidelines.

Before starting the intervention, the teachers of the experimental group were asked to film themselves teaching to evaluate the initial individual motivational style. Subsequently, they received training in an Autonomy Support Intervention Program (PIAA) ( Moreno-Murcia *et al.*, 2019a; Huéscar *et al.*, 2022) for 40 hours, which included several face-to-face sessions aimed at understanding the motivational style of autonomy support and being able to transfer it to the context of their classes effectively. The training took place in two phases from October to December and combined face-to-face and virtual teaching. In the former, theoretical seminars were held, interspersed with practical workshops, in which the models and strategies shown in the literature to implement more autonomous styles and differentiate them from controllers were analysed ( Niemiec and Ryan, 2009; Reeve 2009, 2016; Reeve and Halusic 2009; Reeve and Jang, 2006; Reeve and Cheon, 2015). Videos in which teachers applied strategies were shown and analysed. The aim was to identify the key points of the strategies presented and to establish consensus on their implementation. Group discussion was used to increase the degree of reliability and validity of the measure among participants. This phase was complemented with synchronous and asynchronous virtual training.

In the second phase, based on the proposal of Moreno-Murcia *et al.* (2021) and Huéscar *et al.* (2022), the autonomy support strategies were applied in a graded manner through practical workshops. Thus, the week prior to the implementation of each strategy, the teachers of the experimental group analysed them and wrote examples for their subject, which were reviewed and approved by the group of experts. For training in the autonomy-supportive motivational style, the Measuring Interpersonal Teaching Style (MEID) scale was used ( Barrachina-Peris *et al.* 2022). The same scale was used during the intervention to analyse the motivational style displayed by the participating teachers (control and experimental). Three moments were recorded throughout the intervention: at the beginning, during its development and at the end. According to the literature ( Sarrazin *et al.*, 2006; Aelterman *et al.*, 2014; Haerens *et al.*, 2013; Reeve and Jang, 2006), the use of a given motivational style was determined when the teacher oriented a minimum of 80% of his/her classroom interactions to its application. Thus, in the experimental group, 80% of the interactions had to be directed to the autonomy-supportive motivational style (implementing the strategies by giving autonomy) while in the control group, this percentage had to represent the controlling teaching intervention (applying the strategies by encouraging control).

The indicators obtained by each group in the study are show in Table 1.

**Table 1.** Record of teacher motivational style interactions obtained during the PIAA.

	Moment 1				Moment 2				Moment 3			
	Intervention group		Control group		Intervention group		Control group		Intervention group		Control group	
	Freq.	%	Freq.	%	Freq.	%	Freq.	%	Freq.	%	Freq.	%
Autonomy support	77	60.63	20	23.26	136	95.77	20	22.47	123	94.61	16	17.70
Control style	37	29.13	54	62.79	3	2.11	53	59.55	2	1.53	52	57.77
Neutral style	13	10.24	12	13.95	3	2.11	16	17.98	5	3.84	22	24.44
Total	127	100	86	100	142	100	89	100	130	100	90	100

*Note.* Freq. (Frequency). At moments two and three the experimental group exceeds 90% in autonomy-supportive interactions while the control group remains below 25% and exceeds 55% of interactions in the controlling style.

### Preliminary analysis

First, to test the homogeneity of the two groups before the intervention, a one-factor analysis of variance was performed. Cronbach’s alpha coefficient was used to verify the internal consistency of each factor. To ensure the homogeneity of all dependent variables, a Levene’s test was performed on the pre-test and post-test. The effect of the intervention was assessed through a 2×2 (group × time) repeated measures analysis (ANOVA). To answer the research questions, a repeated measures ANOVA was conducted with all dependent variables (autonomy supportive style, structure style, controlling style and chaos style; psychological need for autonomy, psychological need for competence, psychological need for relatedness; autonomous motivation and controlling motivation and competencies). Cohen’s d was calculated to estimate the effect size. Data analysis was performed with the SPSS 52.0 statistical package.

## Results

To test the homogeneity of the two groups before the intervention, a one-factor analysis of variance was performed, considering as dependent variables (autonomy support style, structure style, controlling style and chaos style; psychological need for autonomy, psychological need for competence, psychological need for relationship; self-determined motivation, controlling motivation and competence) and as a fixed factor (the group), finding significant differences in the controlling motivation variable (p < .05).

### Intervention effect

The repeated measures analysis ( Table 2) revealed significant differences in the variable support for teacher autonomy in the intervention group, improving in the post-test with respect to the pre-test (p < .05). Autonomous motivation significantly improved in the experimental group (p < .05) in the post-test, as did the basic psychological need for autonomy (p < .05) and relationship (p < .05). In relation to the competencies, the final measure showed a significant improvement in the experimental group (p < .05) with respect to the initial measurement, decreasing its value in the control group. In the control group, significant differences were only obtained in the teaching motivational style variable Chaos (p < .05).

**Table 2.** Repeated measures analysis and Cohen's d.

	Intervention			Control		
	*M*_Pre_	*M*_Post_	*d*	*M*_Pre_	*M*_Post_	*d*
A. support style	4.49	4.93 *	-0.38	4.66	4.84	-0.15
Structure style	5.06	5.26	-0.21	5.19	5.26	-0.09
Control style	3.87	3.75	0.12	4.07	4.37	-0.25
Chaos style	2.68	2.86	-0.15	2.90	3.44 *	-0.46
Autonomy	3.65	3.97 *	-0.41	3.50	3.56	-0.06
Competence	3.84	3.94	-0.13	3.80	3.83	-0.03
Relation	4.20	4.31	-0.14	4.04	3.90	0.18
Self-motivation	5.11	5.61 *	-0.49	5.07	5.06	0
Control motivation	4.17	4.41	-0.26	4.81	4.64	0.19
Competences	5.16	5.72 *	-0.52	5.38	5.30	0.07

*Note.* A. (Autonomy).

* p < .05.

## Discussion

The aim of the study was to test the effect of the motivational style of autonomy support on psychological needs, motivation and competence development in secondary school students. The intervention was carried out for three months and included previous teacher training in autonomy support (PIAA). After analysing the data, a generalized positive effect was observed in the experimental group, which supports the initial hypothesis. Thus, students who received their classes through the autonomy-supportive motivational style presented better indicatorss in the satisfaction of basic psychological needs and self-determined motivation, the results being aligned with previous studies ( Reeve, 2011; Chang *et al.*, 2016; Conesa *et al.*, 2022; Reeve and Cheon, 2021; Hosseini *et al.*, 2022; Aelterman *et al.*, 2014; Cheon and Reeve, 2015; Trigueros, *et al.*, 2019; Fin *et al.*, 2019; Moreno-Murcia and Sánchez-Latorre, 2016; Yew and Wang, 2016; Barkoukis *et al.* 2020).

Regarding basic psychological needs, a significant improvement is observed in the autonomy variable, which is important since, according to De Muynck *et al.* (2017) and Cheon *et al.* (2020), it acts independently on well-being factors ( Baten *et al.*, 2020) and is positively associated with agentic commitment, active participation and persistence in their own learning, producing improvement in the student’s academic performance ( Reeve and Shin, 2020) and well-being ( Santana-Monagas *et al.* 2022a). In this sense, the study reveals the importance of adopting a teaching perspective that contemplates the student’s interests and gives them responsibility so that they feel an active part of the development of the tasks, as already pointed out by some recent studies ( Cuevas *et al.*, 2018; Cook-Sather *et al.*, 2021; Jiang and Zang, 2021; Reeve and Shin, 2020) reaffirming existing contributions in the literature ( Niemiec and Ryan, 2009; Reeve, 2006, 2011, 2016). Although significant improvements on competence and relationship needs were expected in the experimental group, the results obtained could be explained around several factors. Firstly, due to the duration of the intervention and its effects on the teaching behavioural pattern, which could have been insufficient for the teacher to reach a self-regulated mastery of the style ( Huéscar *et al.* 2022), revealing some discrepancies between the perception of the application of the style and reality, as previous studies ( Reeve and Jang 2006; Aguado-Gómez *et al.* 2016) pointed out. In this sense, the studies conducted show the existence of a wide variability around the duration and contents for training in the motivational teaching style ( Su and Reeve 2011; Pérez-González *et al.* 2019) with disparate results in the application of the style. Taking into account the time availability of the intervention and with the aim of facilitating its more effective practical application, the PIAA model was followed ( Moreno-Murcia *et al.*, 2021; Huéscar *et al.*, 2022) since it presents a proposal structured in phases that facilitates its application in practice that has demonstrated its validity in the educational context ( Moreno-Murcia *et al.*, 2019a). Another factor could be related to the degree of group and social cohesion of the experimental groups and the management performed by the teacher in class through his motivational style. It has been shown that group cohesion is positively related to the satisfaction of basic needs, autonomous motivation and involvement in class ( Bosselut *et al.*, 2018) and for this purpose the establishment of task climate and the use of interpersonal styles of support for basic psychological needs are of utmost importance ( Leo *et al.*, 2020), since good cohesion has shown its importance in confidence for the resolution of group tasks and collective efficacy, being key in motivational processes ( Leo *et al.*, 2021). The factors described above may have influenced the results obtained with respect to the structure and relationship mediators and coincide with the findings of Waterschoot *et al.* (2019), which highlights the importance of lesson planning so that students feel an active part and are involved in the teaching and learning process.

In relation to the development of competencies, an improvement was observed in the intervention group with respect to the control group. These results coincide with previous studies that showed that active styles improve motivation and the development of competencies ( Moreno-Murcia *et al.* 2020) and that the autonomy-supportive style positively predicts competencies in adolescent students ( Barrachina-Peris, 2017; Moreno-Murcia *et al.*, 2015). Therefore, the autonomy-supportive motivational style can become a facilitator of active learning ( Reeve, 2006; Reeve and Cheon, 2021), basis on which competency learning is built ( Order ECD/65/2015) and competencies are developed ( Bolivar, 2010; Royal Decree 217/2022; EU, 2018). The study shows a positive relationship between the autonomy-supportive motivational style and self-determined motivation, which is consistent with the results of other works ( Abula *et al.*, 2020; Barkoukis *et al.*, 2020; Fin *et al.*, 2019 and Sánchez-Oliva *et al.*, 2017) that support the transfer of the benefits obtained in class to other personal and social contexts of the student, such application process being a determining factor for the development of competencies ( Bolívar, 2010; European Union, 2018).

Framed in SDT ( Deci and Ryan, 2000; Ryan and Deci, 2000) the study follows the line of works showing that autonomously motivated students present a greater willingness to put more effort into different tasks and a higher perceived competence ( Feng *et al.*, 2019; Mouratidis *et al.*, 2011; Ryan and Frederick, 1997; Vansteenkiste *et al.*, 2018), helping to extrapolate it to different tasks of daily life ( Komarraju and Nadler, 2013; Jiang *et al.*, 2019). In this line, Johansen *et al.* (2023) indicate that an autonomously motivated student is more likely to show emotional engagement that leads them to participate in a more active way in various situations where they have to solve tasks due to involvement and enthusiasm and when a context that supports autonomy is posed, people become actively involved ( Ryan and Deci, 2000; Skinner and Pitzer, 2016).

In terms of pedagogical contributions, this study suggests that providing autonomy support in the classroom is fundamental for fostering students’ competencies, thus expanding the practical implications of autonomy support and its relationships with correlates of motivation. Affirming the validity and usefulness of implementing strategies to support autonomy already validated ( Huéscar *et al.*, 2022) such as: giving students a choice of content among different possibilities, offering level options within the tasks themselves, favouring participation and cooperative work, promoting a positive climate in class, guiding the student towards the search for answers without facilitating the solution to the problems posed or the use of non-controlling language, etc. When students perceive that their autonomy is supported through an optimal learning climate, they are more likely to mobilize their internal motivational resources and decide to engage voluntarily in the different tasks, thus facilitating their perception of competence ( Moreno-Murcia and Barrachina Peris, 2022).

The study has promoted teacher collaboration, revealing it to be a key factor in improving the quality of education in schools. Based on these findings, if similar studies are proposed in the future, the variable teacher collaboration could be further regulated and the impact exerted on the teaching and learning process could be analysed, as pointed out by studies that confirm the positive effect on student academic performance ( Hargreaves, 2019; Reeves *et al.*, 2017; Moolenaar *et al.*, 2012; Main and Bryer, 2005; Goddard *et al.*, 2007; Westheimer, 2008), on teacher motivation and satisfaction and the development of innovative practices ( Kolleck, 2019; Vangrieken *et al.*, 2015; Vangrieken *et al.*, 2017; Donmoyer *et al.*, 2012).

The study has several limitations. On the one hand, the sample size. Despite having 12 groups and 62 students, an increase in the sample would allow comparison of the results at the trans-contextual level. Another limitation is associated with the duration of the intervention. To improve the reliability of the data, longitudinal studies covering longer periods (one or more school years) are proposed in order to analyse the long-term effect, especially on the development of competencies at the end of the basic education stage (6-16 years). Another limiting factor would be associated with the specific context of the application process of the autonomy support strategies and their supervision, which could have minimized the effect that other factors may have had on the results (time of the course, group cohesion, among others), factors that we suggest should be considered in future studies that delve deeper in this direction.

In conclusion, this study supports the results obtained in previous works and opens lines of future work aimed at applying the motivational style of autonomy support using specific training programs (PIAA). The work has shown that, if teachers are trained collaboratively, the quality of teaching is increased and improvements are produced in the development of competencies in secondary education, satisfying basic psychological needs, improving self-determined motivation. This study is the first to investigate how the implementation of autonomy support in an educational context of adolescent students is related to the different competencies through the promotion of autonomous motivation, making it necessary for researchers to highlight the importance of building optimal learning environments that support student autonomy.

## Data Availability

Figshare: Dates of PIIE, https://doi.org/10.6084/m9.figshare.24542071.v1 ( Moreno-Murcia, 2023). Data are available under the terms of the Creative Commons Attribution 4.0 International license (CC-BY 4.0).

## References

[ref1] Abula K Beckmann J He Z : Autonomy support in physical education promotes autonomous motivation towards leisure-time physical activity: Evidence from a sample of Chinese college students. *Health Promot. Int.* 2020;35(1):e1–e10. 10.1093/heapro/day102 30590612

[ref2] Aelterman N Vaanstenkiste M Soenens B : Towards an integrative fine-grained insight in motivating and demotivating teaching styles: The merits of a Circumplex approach. *J. Educ. Psychol.* 2019;111:497–521. 10.1037/edu0000293

[ref3] Aelterman N Vansteenkiste M Van den Berghe L : Fostering a nedd-supportive teaching style: Intervention effects on physical education teacher’s beliefs and teaching behaviors. *J. Sport Exerc. Psychol.* 2014;36(6):595–609. 10.1123/jsep.2013-0229 25602142

[ref4] Aguado-Gómez R Díaz-Cueto M Hernández-Álvarez JL : Apoyo a la autonomía en las clases de educación física: percepción versus realidad. *Revista Internacional de Medicina y Ciencias de la Actividad Física y del Deporte/International Journal of Medicine and Science of Physical Activity and Sport.* 2016;16(62):183–202.

[ref5] Barrachina-Peris J Blasco Mira JE : Análisis del desarrollo de las competencias básicas en el currículum de la Educación Física en la ESO en la Marina Baixa: un estudio de caso. *Apunts. Educación Física y Deportes.* 2012;110:36–44. 10.5672/apunts.2014-0983.es.(2012/4).110.04

[ref6] Barrachina-Peris J : Efecto del apoyo a la autonomía en el enfoque por competencias en educación física [Tesis doctoral, Universidad Miguel Hernández de Elche]. 2017. Reference Source

[ref7] Barrachina-Peris J Moreno-Murcia JA Huéscar E : Design and validation of a scale for measuring motivational teaching style. *Cuadernos de Psicología del Deporte.* 2022;22(1):67–80.

[ref8] Barrachina-Peris J Fin G Moreno-Murcia JA : Mejora de las competencias en educación física: intervención basada en las preferencias de práctica de los estudiantes y en el apoyo a la autonomía. *Revista Ibero-Americana de Estudos em Educaçao. Araraquara.* in press;18.

[ref9] Barkoukis V Chatzisarantis NLD Hagger MS : Effects of a school-based intervention on motivation for out-of-school physical activity participation. *Res. Q. Exerc. Sport.* 2020;92:477–491. 10.1080/02701367.2020.1751029 32643561

[ref10] Baten E Vansteenkiste M De Muynck GJ : How can the blow of math difficulty on elementary school children’s motivational, cognitive, and affective experiences be dampened? The critical role of autonomy-supportive instructions. *J. Educ. Psychol.* 2020;112(8):1490–1505. 10.1037/edu0000444

[ref11] Bolivar A : *Competencias básicas y currículo.* Madrid: Síntesis;2010.

[ref12] Bosselut G Heuzé JP Castro O : Using Exploratory Structure Equation Modeling to validate a new measure of cohesion in the university classroom setting: The University Group Environment Questionnaire (UGEQ). *Int. J. Educ. Res.* 2018;89:1–9. 10.1016/j.ijer.2018.03.003

[ref13] Burgueño R Abós Á Sevil-Serrano J : A Circumplex Approach to (de) motivating Styles in Physical Education: Situations-In-School–Physical Education Questionnaire in Spanish Students, Pre-Service, and In-Service Teachers. *Meas. Phys. Educ. Exerc. Sci.* 2023;1–23.

[ref14] Cents-Boonstra M Lichtwarck-Aschoff A Lara MM : Patterns of motivating teaching behaviour and student engagement: A microanalytic approach. *Eur. J. Psychol. Educ.* 2022;37(1):227–255. 10.1007/s10212-021-00543-3

[ref15] Chang YK Chen S Tu KW : Effect of autonomy support on self-determined motivation in elementary physical education. *J. Sports Sci. Med.* 2016;15(3):460–466. 27803624 PMC4974858

[ref16] Cheon SH Reeve J : A classroom-based intervention to help teachers decrease students’ amotivation. *Contemp. Educ. Psychol.* 2015;40:99–111. 10.1016/j.cedpsych.2014.06.004

[ref17] Cheon SH Reeve J Vansteenkiste M : When teachers learn how to provide classroom structure in an autonomy-supportive way: Benefits to teachers and their students. *Teach. Teach. Educ.* 2020;90:103004. 10.1016/j.tate.2019.103004

[ref18] Comisión Europea: Recomendación del Parlamento Europeo y del Consejo, de 22 de mayo de 2018 sobre las competencias clave para el aprendizaje permanente. 2018.

[ref19] Cohen R Katz I Aelterman N : Understanding shifts in students’ academic motivation across a school year: the role of teachers’ motivating styles and need-base experiences. *Eur. J. Psychol. Educ.* 2022;38:963–988. 10.1007/s10212-022-00635-8 PMC9546755 40479431

[ref20] Cook-Sather A Allard S Marcovici E : Fostering agentic engagement: working toward empowerement and equity through pedagogical partnership. *Int. J. Scholarsh. Teach. Learn.* 2021;15(2). 10.20429/ijsotl.2021.150203

[ref21] Conesa PJ Onandia-Hinchado I Dunabeitia JA : Basic psychological needs in the classroom: A literature review in elementary and middle school students. *Learn. Motiv.* 2022;79:101819. 10.1016/j.lmot.2022.101819

[ref22] Cuevas R Gacía-Calvo T González J : Necesidades psicológicas báscias, motivación y compromiso en educación física. *Revista de Psicología del Deporte.* 2018;27(1):97–104.

[ref23] De Muynck G-J Vansteenkiste M Delrue J : The effects of feedback valence and style on need satisfaction, self-talk, and perseverence among tennis players: An experimental study. *J. Sport Exerc. Psychol.* 2017;39:67e80. 28253062 10.1123/jsep.2015-0326

[ref24] Deci EL Ryan RM : The “what” and “why” of goal pursuits: Human needs and the self-determination of behavior. *Psychol. Inq.* 2000;11:227–268. 10.1207/S15327965PLI1104_01

[ref25] Deci EL Ryan RM : *Handbook of Self-Determination Research.* New York: The University of Rochester Press;2002.

[ref26] De Jong L Meirink J Admiraal W : School-based collaboration as a learning context for teachers: A systematic review. *Int. J. Educ. Res.* 2022;112:101927. 10.1016/j.ijer.2022.101927

[ref27] Delrue J Reynders B Vande Broek G : Adopting a helicopter-perspective towards motivating and demotivating coaching: A circumplex approach. *Psychol. Sport Exerc.* 2019;40:110–126. 10.1016/j.psychsport.2018.08.008

[ref28] Donmoyer R Yennie-Donmoyer J Galloway F : The search for connections across principal preparation, principal performance, and student achievement in an exemplary principal preparation program. *J. Res. Leadersh. Educ.* 2012;7(1):5–43. 10.1177/1942775112440631

[ref29] Escriva-Boulley G Tessier D Ntoumanis N : Need-supportive professional development in elementary school physical education: Effects of a cluster-randomized control trial on teachers’ motivating style and student physical activity. *Sport Exerc. Perform. Psychol.* 2018;7(2):218–234. 10.1037/spy0000119

[ref30] Escriva-Boulley G Guillet-Descas E Aelterman N : Adopting the Situation in School Questionnaire to examine physical education teacher’s motivating and demotivating styles using a circumplex approach. *Res. Public Health.* 2021;18:7342. 10.3390/ijerph18147342 34299793 PMC8304182

[ref31] Feng X Xie K Gong S : Effects of parental autonomy support and teacher support on middle school students’ homework effort: Homework autonomous motivation as mediator. *Front. Psychol.* 2019;10:612. 10.3389/fpsyg.2019.00612 30971977 PMC6445893

[ref32] Fin G Moreno-Murcia JA León J : Interpersonal autonomy support style and its consequences in physical education classes. *PLoS ONE.* 2019;14(5):e0216609. 10.1371/journal.pone.0216609 31107894 PMC6527226

[ref33] Franco E González-Peño A Trucharte P : Challenge-based learning approach to teach sports: exploring perceptions of teaching styles and motivational experiences among student teachers. *J. Hosp. Leis. Sport Tour. Educ.* 2023;32:100432. 10.1016/j.jhlste.2023.100432

[ref34] Gillet N Rosnet E Vallerand RJ : Développement d’une échelle de satisfaction des besoins fondamentaux en contexte sportif. *Canadian Journal of Behavioural Science/Revue canadienne des sciences du comportement.* 2008;40(4):230–237. 10.1037/a0013201

[ref35] Goddard YL Goddard RD Tschannen-Moran M : A theoretical and empirical investigation of teacher collaboration for school improvement and student achievement in public elementary schools. *Teach. Coll. Rec.* 2007;109(4):877–896. 10.1177/016146810710900401

[ref36] Gómez Rijo A Jiménez-Jiménez F Fernández Cabrera JM : El apoyo a la autonomía en Educación Física en Educación Primaria desde la percepción del profesorado. *Retos.* 2023;48:575–583. 10.47197/retis.v48.97484

[ref37] Haerens L Aelterman N Van der Berghe L : Observing physical education teacher’s need-supportive interactions in classroom settings. *J. Sport Exerc. Psychol.* 2013;35:3–17. 10.1123/jsep.35.1.3 23404876

[ref38] Haerens L Aelterman N Vansteenkiste M : Do perceived autonomy-supportive and controlling teaching relate to physical education students’ motivational experiences through unique pathways? Distinguishing between the bright and dark side of motivation. *Psychol. Sport Exerc.* 2015;16(3):26–36. 10.1016/j.psychsport.2014.08.013

[ref39] Hargreaves A : Teacher collaboration: 30 years of research on its nature, forms, limitations and effects. *Teach. Teach.* 2019;25(19):603–621. 10.1080/13540602.2019.1639499

[ref40] Hamodi C Moreno-Murcia JA Martín RB : Medios de evaluación y desarrollo de competencias en educación superior en estudiantes de Educación Física. *Estudios pedagógicos (Valdivia).* 2018;44(2):241–257. 10.4067/S0718-07052018000200241

[ref41] Hosseini FB Ghorbani S Rezaeeshirazi R : Autonomy Support, Needs Satisfaction, Motivation, and Intention to Do Physical Activities in Adolescents: A Validation study. *Int. J. Pediatr.* 2022;10(2):15399–15411.

[ref42] Huéscar E Barrachina-Peris J Moreno-Murcia JA : *En búsqueda de la autonomía en educación física.* Barcelona: Ediciones Octaedro;2022.

[ref43] Jang H Reeve J Halusic M : A new autonomy-supportive way of teaching that increases conceptual learning: Teaching in students’ preferred ways. *J. Exp. Educ.* 2016;84(4):686–701. 10.1080/00220973.2015.1083522

[ref44] Jiang AL Zhang LJ : University teachers’ teaching style and their students’ agentic engagement in EFL Learning in China: A Self-Determination Theory and Achievement Goal Theory integrated perspective. *Front. Psychol.* 2021;12:704269. 10.3389/fpsyg.2021.704269 34177748 PMC8222777

[ref45] Jiang J Vauras M Volet S : Autonomy- supportive and controlling teaching in the classroom: A video-based case study. *Educ. Sci.* 2019;9:1–19. 10.3390/educsci9030229

[ref46] Johansen MO Eliassen S Jeno LM : The bright and dark side of autonomy: How autonomy support and thwarting relate to student motivation and academic functioning. *Front. Educ.* 2023;8:1153647. 10.3389/feduc.2023.1153647

[ref47] Kolleck N : Motivational Aspects of Teacher Motivation. *Front. Educ.* 2019;4:85. 10.3389/feduc.2019.00122

[ref48] Komarraju M Nadler D : Self-efficacy and academic achievement: Why do implicit beliefs, goals, and effort regulation matter? *Learn. Individ. Differ.* 2013;25:67–72. 10.1016/j.lindif.2013.01.005

[ref49] Leo FM Mouratidis A Pulido JJ : Perceived teachers’ behavior and students’ engagement in physical education: The mediating role of basic psychological needs and self-determined motivation. *Phys. Educ. Sport Pedagog.* 2020;27:59–76. 10.1080/17408989.2020.1850667

[ref50] Leo FM López-Gajardo MA Fernández-Río J : Desarrollo de la cohesión y las relaciones sociales en clase para optimizar la motivación e implicación en educación física. García-González L , coord. *Cómo motivar en Educación Física.* Servicio de Publicaciones. Universidad de Zaragoza;2021;131–145.

[ref51] León J Domínguez E Núñez JL : Traducción y validación de la versión española de la Échelle de Satisfacción des Besoins Psychologiques en el contexto educativo. *Anales de Psicología/Annals of Psychology.* 2011;27(2):405–411.

[ref52] Ley Orgánica: de 3 de mayo, de Educación. 2/2006.

[ref53] Ley Orgánica: de 9 de diciembre, para la mejora de la calidad educativa. 8/2013.

[ref54] Ley Orgánica: de 29 de diciembre, por la que se modifica la Ley Orgánica 2/2006, de 3 de mayo, de Educación. 3/2020.

[ref55] Main K Bryer F : What does a ‘good’ teaching team look like in a middle school classroom? Bartlett B Bryer F Roebouck D , editors. *Stimulating the “action” as participants in participatory research. Proceedings of the 3rd International Conference on Cognition.* Language, and Special Education;2005.

[ref56] Ministerio de Educación y Formación Profesional: Datos y cigras. Curso Escolar 2021/2022. Secretaría General Técnica. Gobierno de España. 2021.

[ref57] Moé A Consiglio P Katz I : Exploring the circumplex model f motivating and demotivating teaching styles: the role of teacher need satisfaction and need frustration. *Teach. Teach. Educ.* 2022;118(103823):103823. 10.1016/j.tate.2022.103823

[ref58] Monarca H Rappoport S : *Investigación sobre los procesos de cambio educativo: El caso de las competencias básicas en España.* Ministerio de Educación;2013.

[ref59] Moolenaar NM Sleegers PJC Daly AJ : Teaming up: Linking collaboration networks, collective efficacy, and student achievement. *Teach. Teach. Educ.* 2012;28(2):251–262. 10.1016/j.tate.2011.10.001

[ref60] Moreno-Murcia JA : Dates of PIIE.Dataset. *figshare.* 2023. 10.6084/m9.figshare.24542071.v1

[ref61] Moreno-Murcia JA Barrachina-Peris J : Motivar en educación física. No lo dudes aplica la ciencia. Barcelona. Inde. 2022.

[ref62] Moreno-Murcia JA Ferriz A Barrachina-Peris J : *Estilos motivadores docentes y directividad en Educación Física.* Apunts. Educación Física y Deportes;2023.

[ref63] Moreno-Murcia JA Huéscar E Cervelló E : Prediction of adolescents doing physical activity after completing secondary education. *Span. J. Psychol.* 2012;15(1):90–100. 10.5209/rev_SJOP.2012.v15.n1.37288 22379700

[ref64] Moreno-Murcia JA Huéscar E Nuñez JL : Quasi-experimental study protocol to promote an interpersonal style of autonomy support in physical education teachers. *Cuadernos de Psicología del Deporte.* 2019a;19(2):83–101. 10.6018/cpd.337761

[ref65] Moreno-Murcia JA Ruiz M Vera JA : Predicción del soporte de autonomía, los mediadores psicológicos y la motivación académica sobre las competencias básicas en estudiantes adolescentes. *Revista de Psicodidáctica.* 2015;20(2):359–376. 10.1387/RevPsicodidact.11655

[ref66] Moreno-Murcia JA Sánchez-Latorre F : Efectos del soporte de autonomía en clases de educación física. *RICYDE. Revista Internacional de Ciencias del Deporte.* 2016;12:79–89. 10.5232/ricyde2016.04305

[ref67] Moreno-Murcia JA Huéscar E Nuñez JL : Protocolo de estudio cuasi-experimental para promover un estilo interpersonal de apoyo a la autonomía en docentes de educación física. *Cuadernos de Psicología del Deporte.* 2019b;19(2):83–101. 10.6018/cpd.337761

[ref68] Moreno-Murcia JA Llorca-Cano M Huéscar E : Teaching Style, Autonomy Support and Competences in Adolescents. *Revista Internacional de Medicina y Ciencias de la Actividad Física y el Deporte.* 2020;20(80):563–576. 10.15366/rimcafd2020.80.007

[ref69] Moreno-Murcia JA Barrachina-Peris J Ballester Campillo M : Proposal for Modeling Motivational Strategies for Autonomy Support in Physical Education. *Int. J. Environ. Res. Public Health.* 2021;18(14):7717. 10.3390/ijerph18147717 34300167 PMC8307489

[ref70] Mouratidis A Vansteenkiste M Sideridis G : Vitality and interest-enjoyment as a function of class-to-class variation in need-supportive teaching and pupils’ autonomous motivation. *J. Educ. Psychol.* 2011;103:353–366. 10.1037/a0022773

[ref71] Ntoumanis N Standage M : Motivation in physical education classes: A self-determination theory perspective. *Theory Res. Educ.* 2009;7(2):194–202. 10.1177/1477878509104324

[ref72] Niemiec CP Ryan RM : Autonomy, competence and relatedness in the classroom: applying self-determination theory to educational practice. *Theory Res. Educ.* 2009;7(32):133–144. 10.1177/1477878509104318

[ref73] Núñez Alonso JL Martín-Albo Lucas J Navarro Izquierdo JG : *Validación de la versión española de la Échelle de Motivation en Éducation.* Psicothema;2005.

[ref74] OECD: *Definition and Selection of Competencies: Theoretical and Conceptual Foundations.* OCDE;2002.

[ref75] OECD: *The definition and Selection of key Competencies. Executive Summary.* OCDE;2005.

[ref76] OECD: *PISA 2018 Results (Volume I): What Students Know and Can Do.* Paris: PISA, OECD Publishing;2019. 10.1787/5f07c754-en

[ref77] Orden: de 21 de enero, por la que se describen las relaciones entre las competencias, los contenidos y los criterios de evaluación de la educación primaria, la educación secundaria obligatoria y el bachillerato. Ministerio de Educación, Cultura y Deporte. ECD/65/2015.

[ref78] Pérez-Pueyo A (coord.) Casado OM Heras C : *Programar y evaluar competencias básicas en 15 pasos.* Barcelona: Grao;2013.

[ref79] Pérez-González AM Valero-Valenzuela A Moreno-Murcia JA : Revisión sistemática del apoyo a la autonomía en educación física. *Apunts Educación Física y Deportes.* 2019;35(138):51–61. 10.5672/apunts.2014-0983.es.(2019/4).138.04

[ref80] Real Decreto: de 29 de marzo, por el que se establece la ordenación y las enseñanzas mínimas de la Educación Secundaria Obligatoria. Ministerio de Educación y Formación Profesional. 217/2022.

[ref81] Reeve J : Teachers as facilitators: what autonomy-supportive teachers do and why their students Benefit. *Elem. Sch. J.* 2006;106(3):225–236. 10.1086/501484

[ref82] Reeve J : How do I motivate others? The concept of motivating style. *Motivacion y Emocion: Investigaciones Actuales.* 2010;15–28.

[ref83] Reeve J : Why teachers adopt a controlling motivating style toward students and how they become more autonomy supportive. *Educ. Psychol.* 2009;44(3):159–175. 10.1080/004615220903028990

[ref84] Reeve J : Teaching in ways that support students’ autonomy. Mashek D Hammer EY , editors. *Empirical research in teaching and learning: Contributions from social psychology.* Wiley Blackwell;2011; pp.90–103. 10.1002/978144395341.ch5

[ref85] Reeve J : Autonomy-supportive teaching: What it is, how to do it. *Building Autonomous Learners.* 2016; pp.129–152. 10.1007/978-981-287-630-0_7

[ref86] Reeve J Cheon SH : Teachers become more autonomy supportive after they believe it is easy to do. *Psychol. Sport Exerc.* 2015;22:178–189. 10.1016/j.psychosport.2015.08001

[ref87] Reeve J Cheon SH : Autonomy-supportive teaching: Its malleability, benefits, and potential to improve educational practice. *Educ. Psychol.* 2021;56(1):54–77. 10.1080/00461520.2020.1862657

[ref88] Reeve J Halusic M : How K-12 teachers can put self-determination theory principles into practice. *Theory Res. Educ.* 2009;7(2):145–154. 10.1177/1477878509104319

[ref89] Reeve J Jang H : What teachers say and do to support students’ autonomy during a learning activity. *J. Educ. Psychol.* 2006;98(1):209–218. 10.1037/0022-0663.98.1.209

[ref90] Reeve J Shin S : How teachers can support students’ agentic engagement. *Theory Pract.* 2020;59(2):150–161. 10.1080/00405841.2019.1702451

[ref91] Reeves PM Pun WH Chung KS : Influence of teacher collaboration on job satisfaction and student achievement. *Teach. Teach. Educ.* 2017;67:227–236. 10.1016/j.tate.2017.06.016

[ref92] Ryan RM Deci EL : La Teoría de la Autodeterminación y la Facilitación de la Motivación Intrínseca, el Desarrollo Social, y el Bienestar. *Am. Psychol.* 2000;55(1):68–78. 10.1037/0003-066X.55.1.68 11392867

[ref93] Ryan RM Deci EL : *Self-determination theory: Basic psychological needs in motivation, development, and wellness.* Guilford Publications;2017.

[ref94] Ryan R Deci E : Intrinsic and extrinsic motivation from a self-determination theory perspective: definitions, theory, practices and future directions. *Contemp. Educ. Psychol.* 2020;61:101860. 10.1016/j.cedpsych.2020.101860

[ref95] Ryan RM Frederick C : On energy, personality, and health: Subjective vitality as a dynamic reflection of well-being. *J. Pers.* 1997;65:529–565. 10.1111/j.1467-6494.1997.tb00326.x 9327588

[ref96] Sánchez-Oliva D Pulido-Gonzalez JJ Leo FM : Effects of an intervention with teachers in the physical education context: A Self-Determination Theory approach. *PLoS One.* 2017;12(12):e0189986. 10.1371/journal.pone.0189986 29284027 PMC5746241

[ref97] Santana-Monagas E Nuñez JL Loro JF : Teachers’ engaging messages: the role of percieved autonomy, competence and relatedness. *Teach. Teach. Educ.* 2022a;109:103556. 10.1016/j.tate.2021.103556

[ref98] Santana-Monagas E Nuñez JL Loro JF : What makes a student feel vital? Links between teacher-student relatedness and teachers’ engaging messages. *Eur. J. Psychol. Educ.* 2022b;38:1201–1226. 10.1007/s10212-022-00642-9

[ref99] Santana-Monagas E Putwain D Nuñez JL : ¿Predicen los mensajes del profesorado la motivación para aprender y el rendimiento? *Revista de Psicodidáctica.* 2021;27:86–95. 10.1016/j.psioe2021.11.001

[ref100] Sarrazin PG Tessier DP Pelletier LG : The effects of teachers’ expectations about students’ motivation on teachers’ autonomy-supportive and controlling behaviors. *Int. J. Sport Exerc. Psychol.* 2006;4(3):283–301. 10.1080/1612197X.2006.9671799

[ref101] Skinner EA Pitzer JR : Developmental dynamics of student engagement, coping, and everyday resilience. *Handbook of research on student engagement.* Christenson SL , editor. Berlin: Springer Science;2016.

[ref102] Schuster J Hartmann U Kolleck N : Teacher collaboration networks as a function of type of collaboration and schools’ structural environment. *Teach. Teach. Educ.* 2021;103:103372. 10.1016/j.tate.2021.103372

[ref103] Su YL Reeve J : A meta-analysis of the effectiveness of intervention programs designed to support autonomy. *Educ. Psychol. Rev.* 2011;23:159–188. 10.1007/s10648-010-9142-7

[ref104] Taylor I Ntoumanis N Standage M : Motivational predictors of physical education students’ effort, exercise intentions, and leisure-time physical activity: A multilevel linear growth analysis. *J. Sport Exerc. Psychol.* 2010;32(1):99–120. 10.1123/jsep.32.1.99 20167954

[ref105] Tessier D Sarrazin P Ntoumanis N : The effect of an intervention to improve newly qualified teachers’ interpersonal style, students motivation and psychological need satisfaction in sport-based physical education. *Contemp. Educ. Psychol.* 2010;35(4):242–253. 10.1016/j.cedpsych.2010.05.005

[ref106] Tian L Shen J : The effect of perceived teachers’ interpersonal behavior on students’ learning in physical education: a sistemati review. *Front. Psychol.* 2023;14:1233556. 10.3389/fpsyg.2023.1233556 37720632 PMC10499622

[ref107] Trigueros R Mínguez LA González-Bernal JJ : Influence of teaching style on physical education adolescents’ motivation and health-related lifestyle. *Nutrients.* 2019;11(11):2594. 10.3390/nu11112594 31671742 PMC6893640

[ref108] Unión Europea: Recomendación del Consejo, de 22 de mayo de 2018, relativa a las competencias clave para el aprendizaje permanente. 2018. Reference Source

[ref109] Valle J Manso J : Competencias clave como tendencia de la política educativa supranacional de la Unión Europea. *Revista de Educación.* 2013;12–33. 10.44381/1988-592X-RE-2013-EXT-255

[ref110] Vangrieken K Dochy F Raes E : Teacher collaboration: A systematic review. *Educ. Res. Rev.* 2015;15:17–40. 10.1016/j.edurev.2015.04.002

[ref111] Vangrieken K Meredith C Packer T : Teacher communities as a context for professional development: A systematic review. *Teach. Teach. Educ.* 2017;61:47–59. 10.1016/j.tate.2016.10.001

[ref112] Vansteenkiste M Aelterman N Muynck GD : Fostering personal meaning and self-relevance: A self-determination theory perspective on internalization. *J. Exp. Educ.* 2018;86:30–49. 10.1080/00220973.2017.1381067

[ref113] Van Doren N De Cocker K Flamant N : Observing physical education tearcher’s need-supportive and need thwarting styles using a circumplex approach: how does it relate to student outcomes? *Phys. Educ. Sport Pedagog.* 2023;1–25. 10.1080/17408989.2023.2230256

[ref114] Vermote B Aelterman N Beyers W : The role of teachers’ motivation and mindsets in predicting a (de) motivating teaching style in higher education: a circumplex approach. *Motiv. Emot.* 2020;44:270–294. 10.1007/s11031-020-09827-5

[ref115] Vermote B Vansteenkiste M Aelterman N : Teacher’s psychological needs link social pressure with personal adjustment and motivating teaching style. *J. Exp. Educ.* 2022;91:696–717. 10.1080/00220973.2022.2039584

[ref116] Westheimer J : Learning among colleagues: Teacher community and the shared enterprise of education. Cochran-Smith M Feiman-Nemser S McIntyre J , editors. *Handbook of research on teacher education.* Reston, VA and Lanham, MD: Association of Teacher Educators and Rowman;2008; pp.756–782.

[ref117] Waterschoot J Vansteenkiste M Soenens B : The effects of experimentally induced choice on elementary school children’s intrinsic motivation: The moderating role of indecisiveness and teacher-student relatedness. *J. Exp. Child Psychol.* 2019;188:104692. 10.1016/j.jecp.2019.104692 31539835

[ref118] Yew M Wang K : The effectiveness of an Autonomy-Supportive Teaching Structure in Physical Education. *RIDYCE. Revista Internacional de Ciencias del Deporte.* 2016;12(43):5–28.

[ref119] Zang Z : Towards the role of teacher empathy in student’s engagement in english language classes. *Front. Psychol.* 2022;13:880935. 10.3389/fpsyg.2022.880935 35719575 PMC9201024

[ref120] Zapatero-Ayuso JA González-Ribera MD Campos-Izquierdo A : Dificultades y apoyos para enseñar por competencias en educación física en secundaria: un estudio cualitativo. *RICYDE. Revista Internacional de Ciencias del Deporte.* 2017;13:5–25. 10.5232/ricyde2017.04701

