# Supplementary material for: Development of competencies in secondary education through the motivational style of autonomy support
Source: F1000Res. 2024 Mar 7;13:159. [Version 1] doi: 10.12688/f1000research.144919.1 (PMC11380078; doi:10.12688/f1000research.144919.1)
Supplement: Peer review history

# Reviewer report: Reviewer response for version 1

**DOI:** 10.5256/f1000research.158786.r273445

**Published:** 2024-09-06

**Authors:** Gavin R. Slemp, Andrew Napier

**Affiliations:** 1 The University of Melbourne, Melbourne, Victoria, Australia; 2 Centre for Wellbeing Science, The University of Melbourne, Melbourne, Victoria, Australia

I completed this review with the with a PhD student. We reviewed the article separately, and I include my comments first and their comments after mine:

 My comments:

 The aim of the present study was to examine the effect of an intervention based on motivating styles and SDT to see if it yielded beneficial outcomes in secondary school students, using a quasi-experimental design. Results suggested some improvements in autonomy support, autonomy, autonomous motivation, whereas these were generally not observed in the control group.  Authors conclude that they found a positive effect of their intervention.

 Overall, there are some things to like about this paper. It is good we are seeing more interventions like the current study and the intervention seems to be based on solid foundations covered in the SDT literature. That said, I do have some concerns that temper my enthusiasm, which I also outline below. 

 - The introduction gives a lot of background information about SDT and related concepts, such as the circumplex model and motivation subtypes, but it does little to problematize the need for the study. I think you could do more here as there is only a few lines of text at the end of the introduction to say a few interventions exist.  Why do we need more interventions? What will they contribute to knowledge? I think more could be done to elaborate on these points.

 - After reading the method, it was not clear to me how participants were allocated to conditions, and only several pages later did we learn it was a quasi-experimental trial. Please make this important point more salient early in the paper.

 - Not much information is really provided about the intervention. Can you please be clearer about what participants experienced? How many sessions were involved and for how long? What was the focus of each session? Why was the intervention design in the way it was? etc. There is some information provided here but it could be considerably more substantial to give complete information. 

 - Please include a consort diagram that shows the flow of participants from screening all the way through to last measurement. It is useful for readers to understand the extent and effect of attrition. 

 - In preliminary analyses and results sections, the first 2 sentences of each section are verbatim. I would suggest rephrasing given each section should be incorporating different information, which would allow you to better distinguish the preliminary analyses from the actual results presented later.

 - Refer to measurements as time 1, time 2, and time 3, rather than moments, which is the current language used to describe them. Also, frequency should be N? It is not really clear whether table 1 includes N, which makes it very confusing.  Please also ensure to report means, SDs, N for each time point for each group for each variable.

 - Report the actual p-values, rather than thresholds as they are presented in the section "Intervention Effect". Please also give more information, such as the means of both groups and an effect size that compares/quantifies the group by time interactions, which will allow readers to better understand how strong these effects are. Also, a problem with the results section, as well as the paper overall, is that it is generally unclear who the intervention group is. Is it the group who received the training? Or is it the group who studied under those who received the training? I think it would be beneficial to provide two sets of metrics to capture the different layers/cohorts for your intervention effects. 

 - A clear limitation that has not been mentioned is the fact that this study was a quasi-experiment rather than a randomized controlled trial, limiting inferences about causality.

 PhD student:

- The 5 ^th^ paragraph (pg. 3), when discussing the circumplex model, has a typo. It says, “demanding and demanding”, when this should say “domineering and demanding”
- Definition of autonomy on pg. 4 is not accurate. Autonomy is different from independence. Suggest correcting this.
- Suggest also using the correct term “amotivation” rather than “demotivation” (pg. 4)
- The justification for the current study (pg. 5) was fairly short and abrupt. It was unclear what this study offers that previous autonomy-supportive school-based programs have not already investigated. I would suggest giving some extra information regarding this. The use of a clear hypothesis would help clarify the aims of the study.
- It’s a bit unclear why this is a quasi-experimental design. Why were students and teachers not randomly assigned into control/experimental groups? The participant section simply states that they were divided into groups. This doesn’t seem to preclude randomisation. Authors should clarify.
- At the bottom of page six it states that “The sample was distributed into 13 groups, of which 7 had a teacher who followed an intervention model with a motivational style of autonomy support and the remaining 6 did not follow differentiated methodological guidelines”. Should this say 5 not 6 teachers, given in the methods section, only 5 were in the control group.
- I commend the authors on the use of the MEID to measure the autonomy and controlling styles of teachers during the PIAA training. Was this process done blinded or by the researchers who knew which condition the teachers were in? If not blinded, this may lead to bias in rating the experimental group teachers as more autonomy supportive. I would recommend clarifying how this was done and any biases that might affect the rating of teachers motivational style.
- Under Intervention Effect it states: “In relation to the competencies, the final measure showed a significant improvement in the experimental group (p < .05) with respect to the initial measurement, decreasing its value in the control group.” I suggest changing wording around ‘decreasing its value in the control group’ as this sounds as though competences decreased significantly in the control group, when according to table 2 they did not.
- Given the large number of dependent variables that may be related, would it be advised to perform a MANOVA to see if any significant omnibus effects are present before exploring each individual variable
- Limitations of the present study should also mention the lack of randomisation and need for true RCT in the future.

Is the work clearly and accurately presented and does it cite the current literature?

Partly

If applicable, is the statistical analysis and its interpretation appropriate?

Partly

Are all the source data underlying the results available to ensure full reproducibility?

Yes

Is the study design appropriate and is the work technically sound?

Yes

Are the conclusions drawn adequately supported by the results?

Yes

Are sufficient details of methods and analysis provided to allow replication by others?

Partly

Reviewer Expertise:

SDT, motivation, psychology, wellbeing

We confirm that we have read this submission and believe that we have an appropriate level of expertise to confirm that it is of an acceptable scientific standard, however we have significant reservations, as outlined above.

# Reviewer report: Reviewer response for version 1

**DOI:** 10.5256/f1000research.158786.r261426

**Published:** 2024-08-30

**Authors:** Pedro Saenz-Lopez Bunuel

**Affiliations:** 1 Universidad de Huelva, Huelva, Andalusia, Spain

-An approval status which summarizes your views on the article and helps directly determine whether an article will complete peer review.

 The manuscript has the structure is right, the sample is good enough, the analysis is adequate, the references are updated.

 -A peer review report which supports your answers and approval status, with more detail where necessary. Please also include one or two sentences to summarize the article.

 The objective of this article is to measure the effect of an intervention in secondary school students. The design is a quasi-experimental study in which the experimental group develop a motivational style of autonomy support. The tested variables have been satisfaction of basic psychological needs, motivation, and key competencies. the findings demonstrate a positive impact of the supportive motivational style on the satisfaction of the basic psychological need for autonomy, autonomous motivation, and competencies.

 In the study some limitations are shown such as the sample size or the duration of the intervention. They suggest this and other aspects to be considered in future studies.

 Some details to improve the manuscript:

 - INTRODUCTION

 -Competency-based education and motivation

 1st paragraph. Erratum: It gives (It gives)

 -REFERENCES

 Add doi  link and references (if it is possible). For example:

 [1]

 -INTRODUCTION AND DISCUSSION

 The aim is very clear in the abstract. I suggest including more clearly in the text (at the end of the introduction and at the end of the discussion to conclude answering it).

 - INTRODUCTION

 2ND Paragraph. It seems that positive climate depends on emotional aspects (emotional, support, calm down, enthusiastic). After this shows up this sentence: “These data suggest that creating a learning climate in which students feel motivated to complete academic tasks is now a priority for teachers”. It would be necessary to explain the relation between emotional and motivational climate.

- Is the work clearly and accurately presented and does it cite the current literature?

Yes, the manuscript is very clear, and the structure and the information has been presented accurately. Most of the references has been published in the last 6 years

- Is the study design appropriate and does the work have academic merit?

Yes, the aim of the research was to measure the effect of an intervention. Thus, the cuasi-experimental design is appropriate to achieve it.

- Are sufficient details of methods and analysis provided to allow replication by others?

I think so, because the methodology explain the procedure and through some cited references, other researchers could replicate the study.

- If applicable, is the statistical analysis and its interpretation appropriate?

Yes, firstly they have tested the homogeneity of the two groups, then they have verified the internal consistency of each factor and the homogeneity of all variables. Secondly, they have assessed the effect of the intervention and estimate the effect size. Everything through adequate analysis.

- Are all the source data underlying the results available to ensure full reproducibility?

Yes, on the one hand the explanation is clear to reliquiae. On the other hand the data are available through a link

- Are the conclusions drawn adequately supported by the results?

Yes, the findings are clearly shown and the discussion is developed adequately supported by the results of this study and others research's looking for useful conclusions.

Is the work clearly and accurately presented and does it cite the current literature?

Yes

If applicable, is the statistical analysis and its interpretation appropriate?

Yes

Are all the source data underlying the results available to ensure full reproducibility?

Yes

Is the study design appropriate and is the work technically sound?

Yes

Are the conclusions drawn adequately supported by the results?

Yes

Are sufficient details of methods and analysis provided to allow replication by others?

Yes

Reviewer Expertise:

Teaching training

I confirm that I have read this submission and believe that I have an appropriate level of expertise to confirm that it is of an acceptable scientific standard.

## References

[1] : Diseño y validación de una escala observacional sobre el estilo motivador docente. *Cuadernos de Psicología del Deporte* .2022;22(1) : 10.6018/cpd.430321 67-80 10.6018/cpd.430321

# Reviewer report: Reviewer response for version 1

**DOI:** 10.5256/f1000research.158786.r261430

**Published:** 2024-05-07

**Authors:** Estelio H. M. Dantas

**Affiliations:** 1 Universidade Tiradentes, Aracaju, State of Sergipe, Brazil

This review provides an evaluation of the research article titled ‘Development of competencies in secondary education through the motivational style of autonomy support’.

 The article presents a significant contribution to the field of educational psychology, particularly in understanding the role of teaching styles in fostering student motivation and competency development.

 Strengths:

- Rigorous Methodology: The study employs a quasi-experimental design and includes a significant sample of students and teachers, which strengthens the validity of the results.
- Evidence-Based Intervention: The meta-disciplinary intervention based on the autonomy-supportive motivational style is well-grounded in self-determination theory, a recommended practice in educational literature.

Area for Improvement:

- Intervention Detailing: It would be beneficial to provide more details about the specific activities carried out during the intervention to allow for replication in future studies.

Overall, this is a valuable contribution to the field of educational psychology, providing insights into the role of teaching styles in fostering student motivation and competency development. However, further research is needed to fully understand the long-term impacts of such interventions.

Is the work clearly and accurately presented and does it cite the current literature?

Yes

If applicable, is the statistical analysis and its interpretation appropriate?

Yes

Are all the source data underlying the results available to ensure full reproducibility?

Yes

Is the study design appropriate and is the work technically sound?

Yes

Are the conclusions drawn adequately supported by the results?

Yes

Are sufficient details of methods and analysis provided to allow replication by others?

Partly

Reviewer Expertise:

Gerontology, Fitness

I confirm that I have read this submission and believe that I have an appropriate level of expertise to confirm that it is of an acceptable scientific standard, however I have significant reservations, as outlined above.
